# Acidocalcisomes as Calcium- and Polyphosphate-Storage Compartments during Embryogenesis of the Insect *Rhodnius prolixus* Stahl

**DOI:** 10.1371/journal.pone.0027276

**Published:** 2011-11-11

**Authors:** Isabela Ramos, Fabio Gomes, Carolina M. Koeller, Katsuharu Saito, Norton Heise, Hatisaburo Masuda, Roberto Docampo, Wanderley de Souza, Ednildo A. Machado, Kildare Miranda

**Affiliations:** 1 Intituto de Biofísica Carlos Chagas Filho, Universidade Federal do Rio de Janeiro, Rio de Janeiro, Brazil; 2 Instituto de Bioquímica Médica, Universidade Federal do Rio de Janeiro, Rio de Janeiro, Brazil; 3 Diretoria de Programas, Instituto Nacional de Metrologia Normalização e Qualidade Industrial, Xerém, Brazil; 4 Faculty of Agriculture, Shinshu University, Minamiminowa, Nagano, Japan; 5 Department of Cellular Biology, Center for Tropical and Emerging Global Diseases, University of Georgia, Athens, Georgia, United States of America; New Mexico State University, United States of America

## Abstract

**Background:**

The yolk of insect eggs is a cellular domain specialized in the storage of reserve components for embryo development. The reserve macromolecules are stored in different organelles and their interactions with the embryo cells are mostly unknown. Acidocalcisomes are lysosome-related organelles characterized by their acidic nature, high electron density and large content of polyphosphate bound to several cations. In this work, we report the presence of acidocalcisome-like organelles in eggs of the insect vector *Rhodnius prolixus*.

**Methodology/Principal findings:**

Characterization of the elemental composition of electron-dense vesicles by electron probe X-ray microanalysis revealed a composition similar to that previously described for acidocalcisomes. Following subcellular fractionation experiments, fractions enriched in acidocalcisomes were obtained and characterized. Immunofluorescence showed that polyphosphate polymers and the vacuolar proton translocating pyrophosphatase (V-H^+^-PPase, considered as a marker for acidocalcisomes) are found in the same vesicles and that these organelles are mainly localized in the egg cortex. Polyphosphate quantification showed that acidocalcisomes contain a significant amount of polyphosphate detected at day-0 eggs. Elemental analyses of the egg fractions showed that 24.5±0.65% of the egg calcium are also stored in such organelles. During embryogenesis, incubation of acidocalcisomes with acridine orange showed that these organelles are acidified at day-3 (coinciding with the period of yolk mobilization) and polyphosphate quantification showed that the levels of polyphosphate tend to decrease during early embryogenesis, being approximately 30% lower at day-3 compared to day-0 eggs.

**Conclusions:**

We found that acidocalcisomes are present in the eggs and are the main storage compartments of polyphosphate and calcium in the egg yolk. As such components have been shown to be involved in a series of dynamic events that may control embryo growth, results reveal the potential involvement of a novel organelle in the storage and mobilization of inorganic elements to the embryo cells.

## Introduction

Chagas disease is one of the main causes of cardiac lesions in Latin America, and the blood sucking insect *Rhodnius prolixus* is an important vector for this disease [1]. It is generally acknowledged that the ability of insects to inhabit a variety of niches, and thus become vectors of numerous diseases, is partially due to their high reproductive outputs. Some insects are able to lay a mass of eggs equivalent to half of their body mass, and usually more than 95% of the eggs produced are viable [2]. Therefore, studies regarding aspects of the egg's structure and function could result in novel approaches to population control of disease vectors.

In oviparous insects, the process of embryogenesis occurs totally disconnected from the maternal body, requiring the previous storage, during oogenesis, of all the nutritional reserves for the growing embryo. The amino acid reserve in the eggs is mainly represented by the yolk proteins, which usually are large phosphoproteins that can bind lipids and carbohydrates, and are stored in organelles called yolk granules. Yolk consumption starts when the yolk proteins undergo a process of degradation, which occurs by activation of acid hydrolases also stored within yolk granules. To activate the hydrolases, yolk granules are acidified via proton pumps, such as the vacuolar proton ATPase (V-H^+^-ATPase) [3]–[5] and the vacuolar proton pyrophosphatase (V-H^+^-PPase) [6]. In this process, commonly known as yolk mobilization, amino acids and monosaccharides are produced and readily consumed by the embryo cells. In general, yolk degradation occurs in a regulated manner, at a specific time point of the early embryogenesis that may vary depending on the species. In *R. prolixus*, yolk mobilization starts on the third day of embryogenesis, for a total embryonic development of 12–13 days [7].

Although the basic events involved in yolk mobilization have been generally well known (acidification of yolk granules leading to activation of hydrolases followed by degradation of yolk proteins), little is known about how this process is controlled, i.e. how the different organelles from the yolk couple their activities to perform these highly regulated processes. In general, all vesicles extracted from the yolk are referred to as yolk granules. However, it is accepted that the vesicle population in the yolk is not homogeneous. In several species, yolk granules can be fractionated according to their different size and density [3], [8]–[10]. It has also been shown in different species that small vesicles have a particular subset of hydrolases [10], [11], and are mainly distributed in the peripheral cytoplasm (cortex) of the mature egg. In this region, the vesicles are preferentially acidified, and this process is somehow involved in yolk mobilization [12], [13].

Acidocalcisomes are lysosome-related organelles widely distributed in several microorganisms. They are characterized by their acidic nature, high electron density and large content of polyphosphate (poly P) bound to several cations. They are rich in sodium, magnesium, phosphorus, potassium, calcium, iron and zinc [14], [15]. These organelles have a variety of cation and proton pumps, such as the V-H^+^-ATPase and the V-H^+^-PPase, and Na^+^/H^+^ and Ca^2+^/H^+^ exchangers in their membrane. Although first and better described in trypanosomatids, acidocalcisomes have also been found in several other microorganisms, including Apicomplexan parasites [16], [17], [18], bacteria [19], [20], the green algae *Chlamydomonas reinhardtii* [21] and the slime mold *Dictyostelium discoideum* [22]. They have been implicated in several functions, including storage of cations, ion homeostasis, osmoregulation and poly P metabolism [23]. In the recent past, the presence of acidocalcisome-related organelles has been described in different cell types from higher eukaryotes, including human platelets, in which poly P from acidocalcisomes was found to act as a modulator of blood clotting [24], [25], and eggs of different animals including the insect *Periplaneta americana* [26], sea urchins [27] and chicken [28]. However, the functional roles of acidocalcisomes in these latter organisms, as integral part of the egg yolk, are still unknown.

In this study, we characterized the presence of acidocalcisome-like organelles in the eggs of *R. prolixus*. Using transmission electron microscopy coupled with X-ray microanalysis, electron dense organelles presenting the typical elemental composition of acidocalcisomes were found in the eggs. These organelles were localized in the egg cortex and presented other typical characteristics of acidocalcisomes such as the presence of a V-H^+^-PPase and poly P. Quantification of inorganic elements showed that at least ∼24% of the total calcium in the egg is present in the acidocalcisomes at day-0 of embryogenesis, thus suggesting that these organelles are an important calcium storage compartment in the eggs. Microscopic analysis of acridine orange stained preparations and poly P quantification at different days of early embryogenesis showed that acidocalcisomes are acidic at day-3 and that the poly P content tends to decrease after day-1 of embryogenesis. Taken together, the results reveal new aspects of early embryogenesis in *R. prolixus*, and the potential involvement of a novel organelle in the storage and mobilization of inorganic components in oviparous animals.

## Methods

### Insects and eggs

*R. prolixus* Stahl, 1859 (Hemiptera, Reduviidae) were reared in a colony maintained at 28°C and 70–80% relative humidity. The insects were fed with rabbit blood in an artificial apparatus as described by Garcia et al. (1975) [29]. Non-fertilized eggs were laid by non-mated adult females and collected 1 h after oviposition. Fertilized eggs were collected and used immediately or allowed to develop until the indicated embryogenesis stage.

### Preparation of total egg homogenates (TEH) and acidocalcisome-enriched fraction (acidocalcisome fraction)

Total egg homogenates (TEH) were prepared by disrupting the eggs, with a plastic pestle, on ice cold buffer containing 10 mM Hepes pH 7.2, 4 mM MgCl_2_, 50 mM KCl, and a protease inhibitors cocktail (Sigma-Aldrich, P-8340).

A fraction enriched in acidocalcisomes was obtained by selectively lysing most classic yolk granules in a hyposmotic buffer (5 mM Hepes, pH 7.2) at room temperature (22°C) for 10 min. Approximately 30 mg of day-0 eggs were disrupted in 500 µl of the hyposmotic buffer described above, supplied with protease inhibitors. The sample was centrifuged twice at 10,000 *g* for 1 min at 4°C in the same buffer, and once in 5 mM Hepes plus 8.5% sucrose. The final pellet was used as acidocalcisome-enriched fraction (acidocalcisome fraction) and was chemically fixed, quickly frozen or resuspended in appropriate buffer for the different assays or procedures as described in the following sections. The supernatant of the first centrifugation (containing the osmotically disrupted yolk granules) was also used in some experiments, and will be referred from now on as yolk fraction.

### Transmission electron microscopy (TEM), X-ray microanalysis and elemental mapping

For conventional transmission electron microscopy (TEM), samples were fixed in freshly prepared 4% formaldehyde, 2.5% glutaraldehyde diluted in 0.1 M sodium cacodylate buffer pH 7.3 at 4°C for 24 h, and then embedded in epoxy resin, sectioned and stained using standard methods. For X-ray microanalysis, the samples were applied onto Formvar-coated copper grids and blotted dry with a filter paper. Samples were examined in a JEOL 1200 EX transmission electron microscope operating at 80 kV. For spectra, X-rays were collected for 100 s using a Si (Li) detector with Norvar window on a 0 to 10 KeV energy range with a resolution of 10 eV/channel. Analyses were performed using a Noran/Voyager III analyzer. For elemental mapping, the images were recorded (256×192 pixels) with a frame time of 50 s and a total of 40 frames (dwell time 1.017 µs) with the EDS pulse processor at rate 2. Analyses were performed using the software NSS 2.3 X-ray Microanalysis (Thermo Fisher Scientific). Semi-quantitative elemental analysis was performed using the Cliff-Lorimer method, as previously described by Miranda et al. (2004) [30]. Briefly, the atomic % was calculated from the measured weight % values (wt. %/ atomic wt.). The sum of the atomic weights of the selected elements was then normalized to 100%. Results were expressed as the percentage of the elements in the spectra. Cation signals were also normalized and represented as the percentage of the phosphorus signal.

### Freeze-fracture

Acidocalcisome fractions were fixed in 2.5% glutaraldehyde and 4% of freshly prepared formaldehyde in 0.1 M cacodylate buffer pH 7.4 for 60 min at room temperature, washed in 0.1 M phosphate buffer pH 7.2, and infiltrated in 30% glycerol. The material was then mounted on aluminum support disks and slammed onto liquid nitrogen-cooled gold block using a quick freezing device (Leica, EM CPC). Fracture was carried out at −115°C in a Balzers-Leica freeze-fracture apparatus (Bal-Tec BAF 060 freeze etching system). Platinum was evaporated onto the specimen at an angle of 15° and carbon was evaporated at an angle of 90°. Replicas were cleaned to remove the remaining organic material by treatment with sodium hypochloride, rinsed with distilled water, mounted on 300-mesh nickel grids and observed in a Zeiss EM 900 transmission electron microscope operating at 80 kV.

### Staining with acridine orange

Acidocalcisome fractions obtained from eggs of different days during early embryogenesis were stained with 6 µM acridine orange for 5–20 min at room temperature in the dark. Images were obtained using a Zeiss Axioplan fluorescence microscope coupled with a charge-coupled device (CCD) camera using appropriate filter sets (λex = 450–490 nm, λem>500 nm).

### Preparation of anti-V-H^+^-PPase polyclonal antibodies

One hundred micrograms of KLH-conjugated synthetic peptide corresponding to the hydrophilic loop IV of plant V-H^+^-PPase (KIATYANARTTLEARKGVGKAFIVAFR) were used to immunize New Zealand white female rabbits using complete Freund's adjuvant for the initial injection and incomplete Freund's for booster injections. Pre-immune serum was tested to monitor the absence of cross-reactivity with the antigenic peptide. Anti-V-H^+^-PPase antiserum was used for western blot and immunofluorescence analyses as described in the following sections.

### Western blot analysis

Sixty micrograms of protein [31] obtained from total cell extracts of *T. cruzi* epimastigote forms, human macrophages (Mø) or from different fractions of day-0 eggs (TEH, acidocalcisome fraction or yolk fraction) were submitted to 10% SDS-PAGE [32] and electrophoretic transfer of the proteins to nitrocellulose membranes was carried out using a Trans-Blot Cell apparatus (Bio-Rad) at 300 V for 2 h at 4°C. The membranes were incubated in blocking buffer containing 10 mM Tris pH 7.2, 150 mM NaCl, 3% (w/v) bovine serum albumin (BSA) and 0.1% (v/v) Tween 20 for 2 h at room temperature. Membranes were then incubated for 3 h with the primary anti-V-H^+^-PPase polyclonal antibodies (1∶1,000) in blocking buffer for 3 h, followed by three washes and incubations with goat anti-rabbit antibody coupled with alkaline phosphatase (1∶5,000) in blocking buffer for 1 h at room temperature. Stain development was carried out using the NBT/ BCIP reaction.

### Immunogold electron microscopy (IEM)

Day-0 eggs were fixed in 0.2% glutaraldehyde, 4% of freshly prepared formaldehyde and 0.5% picric acid in 0.1 M cacodylate buffer pH 7.4 for 24 h at 4°C. After fixation, samples were washed, dehydrated in acetone series (25 min each), and embedded in LR-White resin at 4°C. Polymerization was carried out under UV radiation for 96 h at −20°C. Thin sections of LR-White embedded material were collected on nickel grids, incubated in 100 mM NH_4_Cl in in 50 mM Tris-HCl pH 7.4, 150 mM NaCl (TBS) for 30 min, and transferred to blocking buffer for 30 min at room temperature. Grids were incubated with the primary anti-V-H^+^-PPase antibodies diluted 1∶500 as above. After washing, grids were incubated with 15 nm gold-labeled goat anti-rabbit IgG, diluted 1∶100 in blocking buffer for 1 h at room temperature. The sections were washed, stained in uranyl acetate and lead citrate, and observed in JEOL 1200 EX transmission electron microscope. Quantification of gold particles was performed in random fields from five different sections obtained from three experiments.

### Immunofluorescence analyses using antibodies against the V-H^+^-PPase and the poly P binding domain of exopolyphosphatase

For localization of V-H^+^-PPase and poly P, anti-V-H^+^-PPase antibodies were used together with antibodies against the recombinant poly P-binding domain of *E. coli* exopolyphosphatase linked to an *Xpress* epitope tag (PPBD), as previously described [33]. Acidocalcisome fractions were incubated for 20 min in blocking buffer and for additional 30 min in 12 µg/ml of recombinant PPBD in blocking buffer, washed and fixed in 4% of freshly prepared formaldehyde in 0.1 M cacodylate buffer pH 7.4 for 30 min. After two washes at room temperature, samples were allowed to adhere to poly-L-lysine-coated coverslips and were incubated for 30 min in 100 mM NH_4_Cl and 30 min in blocking buffer. The samples were then incubated with primary anti-V-H^+^-PPase antibodies diluted 1∶300 and 5 µg/ml of anti-Xpress epitope tag (Invitrogen) in blocking buffer for 1 h at room temperature. After washes, secondary antibodies (Alexa Fluor^tm^ 488 goat anti mouse IgG and Alexa Fluor^tm^ 568 goat anti rabbit IgG, Invitrogen) diluted 1∶400 were incubated with the samples for 1 h at room temperature. Immunofluorescence images were obtained using a Zeiss Axioplan fluorescence microscope coupled with a CCD camera using appropriate filter sets (λex_Alexa488_ = 450–490 nm, λem = 510–560 nm and λex_Alexa546_ = 510–525 nm, λem = 560–600) or a Zeiss LSM310 Confocal Microscope equipped with an argon laser.

### Extraction and quantification of long- and short-chain Poly P

TEH, yolk fractions and acidocalcisome fractions were treated with methods to extract either long-chain (LC) or short-chain (SC) Poly P as described by Ault-Riché et al. (2002) [34] and Ruiz et al. (2001) [35], respectively. Poly P levels were determined from the amount of phosphate (P_i_) released upon treatment with an excess of recombinant *S. cerevisiae* exopolyphosphatase 1 (rScPPX1). The recombinant enzyme was prepared as described before [35]. Aliquots of poly P extracts (always less than 1.5 nmol, monomeric P_i_) were incubated for 15 min at 35°C with 60 mM Tris-HCl, pH 7.5, 6 mM MgCl_2_, and 3,000–5,000 units of purified rScPPX1 in a final volume of 100 µl. Release of P_i_ was monitored by the microplate method of Lanzetta et al. (1979) [36]. A standard curve of sodium phosphate was included in all microplate assays and activity towards Poly P_75+_ (Sigma-Aldrich) at a final concentration of 300 nM (in terms of polymer) was included as a control for yield.

### Poly P detection in agarose gels and DAPI staining

Total Poly P was extracted as described before by Gomes et al. (2008) [37]. Acidocalcisome fractions were resuspended in water and sonicated (probe sonication, 30% amplitude, three cycles of 20 s). After treatment with DNase (10 µg/ml) and RNase (10 µg/ml) for 30 min at 37°C, one volume of chloroform was added followed by vortexing for 5 min. The samples were centrifuged at 10,000 *g* for 5 min at 4°C to separate the phases. The water-soluble fraction was collected, dried in a speed-vac, and suspended in 60 mM Tris-HCl pH 7.5, 6 mM MgCl_2_. Poly P samples were then mixed with DNA loading buffer (10 mM Tris-HCl pH 7.5, 10 mM EDTA, 0.25% Orange-G and 0.65% sucrose) and loaded into 1–2% agarose gels. The gels were run at 200 V in Tris-acetate pH 8.2, 1 mM EDTA (TAE buffer) until the dye reached the middle of the gel. Staining was performed as described before by Smith and Morrissey (2007) [38] with minor modifications. Gels were incubated for 30 min in the dark with 2 µg/ml DAPI, 10 mM EDTA and 0.3% Fluoromount-G, and washed twice in the same solution without DAPI for 1 h. Images of DAPI fluorescence were acquired with Alpha Imager gel imaging system (AlphaInotech) using an excitation wavelength of 365 nm.

### Preparation of membrane fractions and PPase activity (PPi hydrolysis)

Yolk fractions and acidocalcisome fractions from day-0 eggs were prepared by differential centrifugation as previously described by Motta et al. (2004) [6], and the PPase activity measured by detecting Pi released from PPi by the method of Fiske and Subbarow (1925) [39] using a microplate reader. The reaction medium contained 50 mM MOPS-Tris pH 7.5, 100 mM KCl, 0.3 mM PPi, and 0.6 mM MgCl_2_. When used, aminomethylenediphosphonate (AMDP) concentration was 40 µM. Reactions were started by the addition of membrane fractions (40 µg protein/ml final concentration) and stopped by the addition of 50% (w/v) trichloroacetic acid after 1 h at 28°C.

### Quantification of elements, inductively coupled plasma optical emission spectroscopy (ICP-OES)

A total of 165 mg of eggs (wet weight) were disrupted and fractionated into acidocalcisome and yolk fractions. The samples were dried on speed-vac and treated with sub-distilled HNO_3_ for 30 min at 80°C. The samples were then diluted in ultra-pure water and read in an inductively coupled plasma optical emission spectrometer (Optima 4300 DV, Perkin Elmer Instruments, Norwalk, CT, USA). The analytical lines used were 213.618 nm for phosphorus (Limit of detection, LOD = 0.030 mg/L, axial view), 393.366 nm for calcium (LOD = 0.0001 mg/L, radial view), 279.553 nm for magnesium (LOD = 0.0002 mg/L, radial view) and 589.592 nm for sodium (LOD = 0.002 mg/L, axial view). The values were obtained by comparing the readings with a calibration curve for each element.

## Results

### Electron dense organelles in the eggs of *R. prolixus*

**Figure 1:**
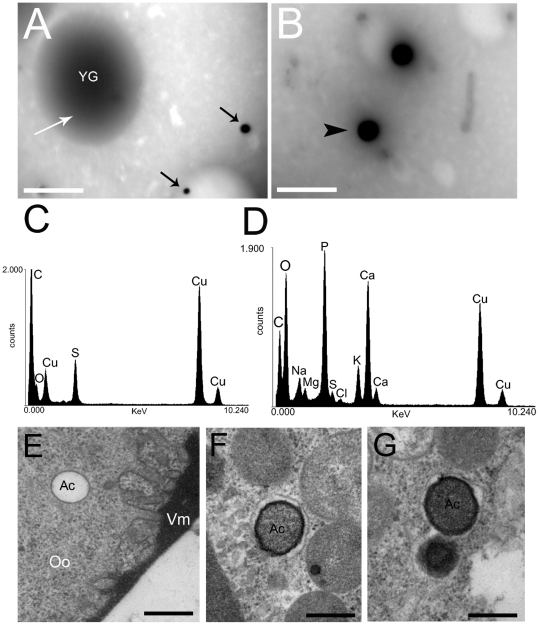
Electron-dense small vesicles in the eggs have the typical elemental composition of acidocalcisomes. **A, B,** Suspensions of eggs organelles were applied to Formvar-coated grids and directly observed in the transmission electron microscope. YG: yolk granule. Black arrows in A: small electron-dense vesicles. Bars: A, 10 µm. B, 1 µm. **C,** X-ray spectrum of the yolk granule shown in A (white arrow). **D,** X-ray spectrum of the electron-dense vesicle pointed in B (arrowhead). **E, F, G,** Standard transmission electron micrographs of the egg cortex showing the presence of empty vesicles and vesicles containing residual electron dense content. Ac: acidocalcisomes. Vm: vitelline membrane. Oo: ooplasm. Bars: 1 µm and 200 nm, respectively.

Day-0 eggs (at cleavage stage, before the beginning of blastoderm, [40]) were disrupted in appropriate buffer and the obtained suspension placed in Formvar-coated grids for direct observation in the transmission electron microscope. Results showed the presence of organelles differing in size and electron density (Figure 1A). X-ray elemental analysis of the larger (>3 µm) and less electron dense vesicles (Figure 1A, white arrow) only presented accumulated sulfur peaks (Figure 1C), suggesting the presence of high amounts of protein. Additionally, several smaller (0.57±0.21 µm) and highly electron dense vesicles were also observed (Figure 1A, black arrows, and B arrowhead). Their elemental profiles were qualitatively similar (for at least 10 X-ray spectra taken) to acidocalcisomes of unicellular eukaryotes [15] showing accumulated sodium, magnesium, phosphorus, chlorine, potassium and calcium (Figure 1D). To further investigate the ultrastructural aspects of these organelles, whole eggs were processed for TEM. Standard procedures revealed the presence of empty vesicles (Figure 1 E), and vesicles containing a residual electron dense content (Figure 1 F, G) (630±190 nm) in the egg cortex, which corresponds to the typical morphology of acidocalcisomes after washing with buffers and organic solvents during the sample fixation/dehydration procedures [41].

### Localization of V-H^+^-PPase

**Figure 2:**
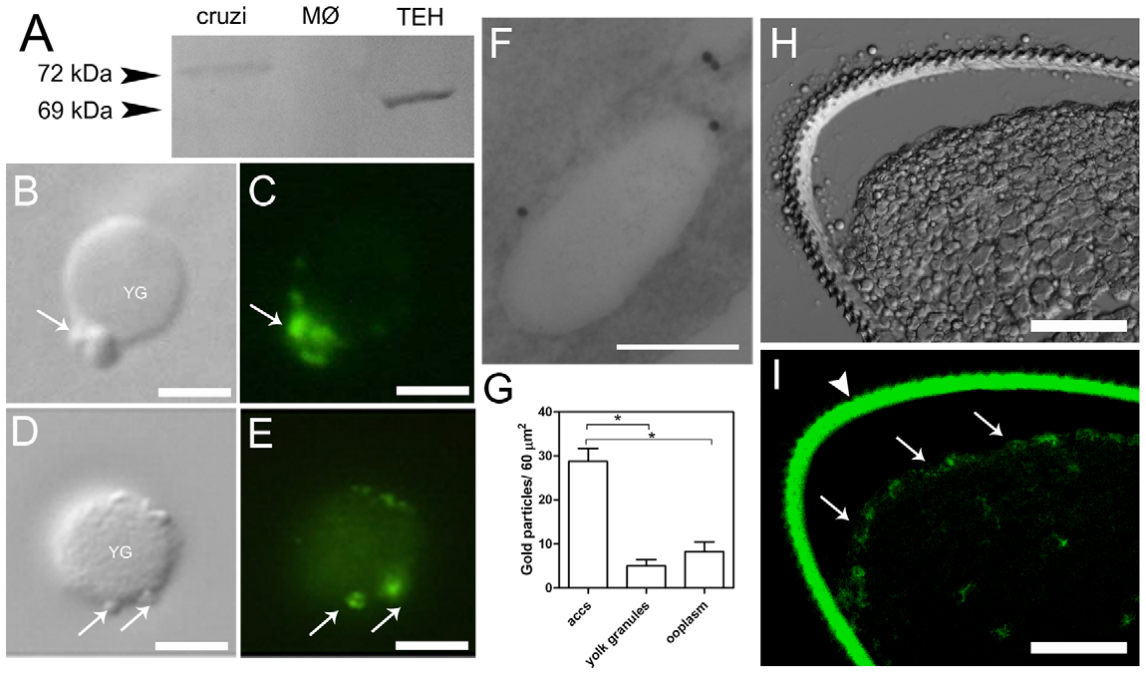
Localization of the vacuolar H^+^-PPase in small vesicles in the egg cortex. **A,** Western blot analysis using anti-V-H^+^-PPase polyclonal antibodies. *Lane cruzi*: *T. cruzi* epimastigotes. *Lane MØ:* human macrophages. *Lane TEH:* total egg homogenates. Arrowheads indicate the relative position of molecular markers in kDa. **B, C, D, E,** Immunofluorescence of the V-H^+^-PPase in the egg organelles adhered to glass slides. Arrows indicate labeled small vesicles. YG: yolk granule. Bars: 10 µm. **F,** IEM of LR-White resin embedded samples showing the localization of the V-H^+^-PPase (gold particles) in the periphery of empty vesicles. Bar: 500 nm. **G,** quantification of gold particles in the different organelles of the egg cortex. Accs: acidocalcisome-like organelles. (*) indicate significant difference (One Way ANOVA, p<0.05). **H, I,** Confocal laser scanning microscopy image (optical section) showing the strong autofluorescence in chorion (white arrow head) and V-H^+^-PPase localization (white arrows) in a thick longitudinal section of the egg. (See also movie S1). Bars: 200 µm.

The V-H^+^-PPase has been previously considered a marker for acidocalcisomes in several microorganisms [15] and Motta et al. (2004) [6] described the presence of this enzyme activity in eggs of *R. prolixus*. Polyclonal antibodies against the conserved loop IV of the V-H^+^PPase from *A. thaliana* were already shown to cross-react with a ∼70 kDa fragment in oocytes of *R. prolixus* [6] and with the V-H^+^-PPase of *T. cruzi* [41], [42], *Leishmania spp.* [43], and *Plasmodium spp.* [17]. For this reason, we decided to investigate the localization of the V-H^+^-PPase in the eggs of *R. prolixus* using anti-V-H^+^-PPase polyclonal antibodies raised in the present study in rabbit. Western blotting using day-0 eggs, *T. cruzi* epimastigotes as a positive and human macrophages as a negative controls, respectively, revealed a positive cross-reaction fragment of ∼69 kDa in *Rhodnius* eggs (Figure 2A). Immunofluorescence in *T. cruzi* epimastigotes confirmed the labeling pattern expected for acidocalcisomes (not shown). Also, immunofluorescence using a suspension of the egg organelles showed that only small vesicles were labeled with anti- V-H^+^-PPase, but not large yolk granules (Figure 2B–E). Immunogold electron microscopy showed that the periphery of the acidocalcisome-like empty vesicles was specifically labeled by the V-H^+^-PPase antibodies (Figure 2F), accumulating a significantly higher amount of gold particles when compared to the other organelles from the egg cortex (Figure 2G). Confocal laser scanning microscopy of thick longitudinal sections of day-0 eggs submitted to immunolocalization assays showed that the V-H^+^-PPase is mainly localized in the egg cortex (Figure 2H and I, white arrows; movies S1 and S2), coincident with the localization of the empty vesicles described in Figures 1E and F. Control experiments (omitting the primary antibodies), did not show labeling in the cortex, although a strong auto fluorescence is seen in the chorion (Figure 2I, white arrow head; movie S1).

### Isolation of the acidocalcisome-like organelles

**Figure 3:**
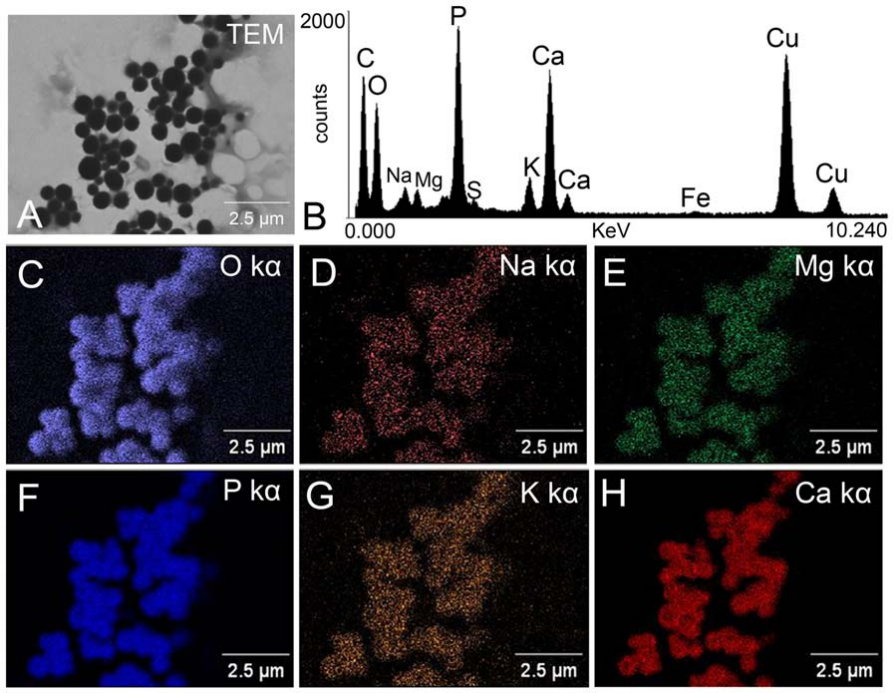
X-ray microanalysis and elemental mapping of isolated acidocalcisome-like organelles. Isolated acidocalcisome-like organelles were analyzed by electron probe X-ray microanalysis. **A.** Transmission electron microscopy image of whole, unfixed, isolated acidocalcisomes adhered to Formvar-coated grids. **B.** X-ray spectrum corresponding to the acidocalcisome indicated by the *arrow* in panel **A.** Copper peaks in the spectrum came from the grid. **C–H.** Elemental mapping of the organelles shown in **A** reveals the co-localization of oxygen, sodium, magnesium, phosphorus, potassium, and calcium. Bars = 2.5 µm.

**Figure 4:**
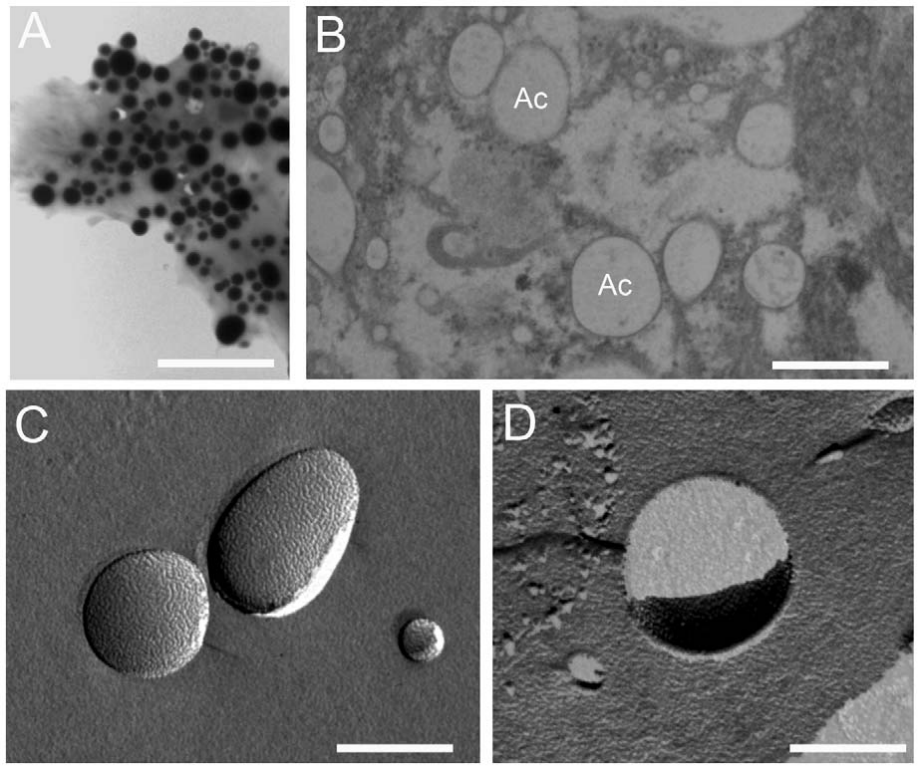
Standard transmission electron microscopy and freeze-fracture of isolated acidocalcisomes. **A,** Transmission electron microscopy of acidocalcisome fractions observed as whole mounts adhered to formvar-coated grids. Bars: 3 µm. **B,** Standard transmission electron microscopy of the chemically fixed acidocalcisomes in the fraction, evidencing the integrity of the acidocalcisome membrane. Ac: acidocalcisomes. Bar: 1 µm. **C, D,** Freeze-fracture of the same fraction showing E- and P- faces of fractured membranes, respectively. Bars: 500 nm.

Differential centrifugation protocols using hypotonic buffers allowed us to selectively disrupt the larger yolk granules and obtain a highly enriched fraction of acidocalcisome-like organelles (acidocalcisome fraction) (Figures 3A). X-ray microanalysis showed that the ion content of the acidocalcisomes in the recovered fraction is retained, showing considerable amounts of oxygen, sodium, magnesium, phosphorus and calcium (Figure 3A and B). X-ray elemental mapping established that these elements are mainly stored in the acidocalcisome-like organelles (Figures 3C–3H). Morphometric analysis showed that the average diameter of the recovered organelles (Figure 4A) (550±260 nm) is similar to the acidocalcisomes found in the suspensions of egg organelles and in thin sections of standard TEM (Figure 1). In addition, standard TEM (Figure 4B) and freeze-fracture (Figures 4C and D) of isolated acidocalcisomes confirmed that the membranes of the organelles are intact after the fractionation procedure.

### V-H^+^-PPase and poly P are localized in the acidocalcisome-like organelles

**Figure 5:**
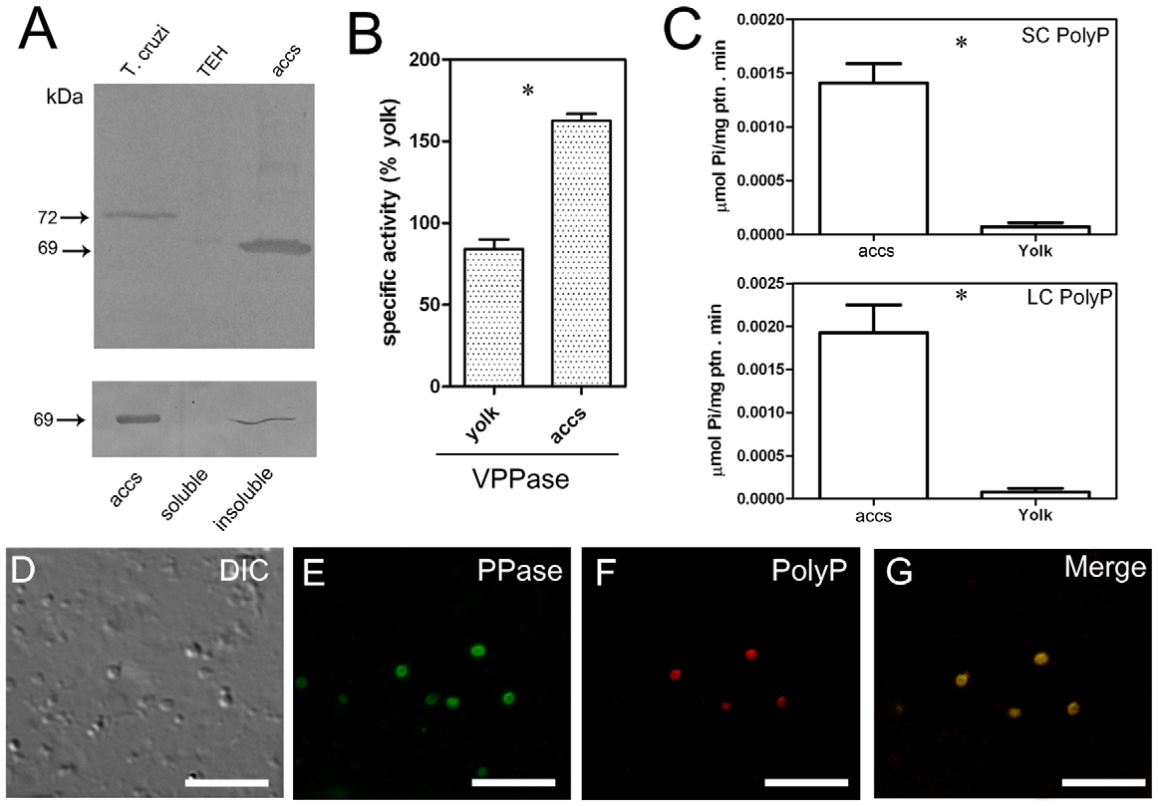
Vacuolar H^+^-PPase and poly P co-localize to the acidocalcisomes. **A,** Upper panel: Western blot analysis using anti-V-H^+^-PPase polyclonal antibody in the total egg homogenate (*lane TEH*) and acidocalcisome-fraction (*lane accs*), showing enrichment of this enzyme in the acidocalcisomes. The extract of *T. cruzi* epimastigote sample (*lane T. cruzi*) was loaded as a positive control for antibody binding. Bottom panel: Acidocalcisome fractions were separated into soluble and insoluble components by differential ultracentrifugation and tested by western blot analysis for V-H^+^-PPase. *Lane accs:* total acidocalcisome fraction. *Lane soluble:* supernatant of acidocalcisome preparation. *Lane insoluble:* pellet of acidocalcisome preparation. All lanes were loaded with 60 µg of protein. **B,** Membrane preparations of yolk and acidocalcisome fractions (accs) were obtained and tested for PPi hydrolysis activity. Data are from 3 experiments, and show means ± S.E.M. (*) indicates significant differences (*t*-test, p<0.05). **C,** Short- and long-chain poly P were extracted and quantified in the yolk and acidocalcisome fraction. Data are from 4 experiments, and show means ± S.E.M. (*) indicates significant differences (t-test, p<0.05). **D, E, F, G,** localization of poly P by immunofluorescence, using an antibody against recombinant poly P binding domain of *E. coli* exopoyphosphatase (PPBD) linked to an *Xpress* epitope tag, and V-H^+^-PPase, using anti-V-H^+^-PPase polyclonal antibodies. Bars: 10 µm.

Western blot analysis using the acidocalcisome fraction showed an increase in immunolabeling for the V-H^+^-PPase when compared to the same amount of loaded protein from yolk fractions (Figure 5A, upper panel). To confirm that the V-H^+^-PPase is associated with the membrane (insoluble) fraction of the organelles, acidocalcisome fractions were frozen and thawed in liquid N_2_, and soluble and insoluble components were separated by ultracentrifugation. Immunodetection by Western blot showed that the enzyme is indeed associated with the insoluble components of the acidocalcisomes (Figure 5A, bottom panel), thus confirming that the V-H^+^-PPase is associated with the organellar membranes. The presence of PPi hydrolysis activity was tested in membrane preparations of the same samples and the results showed that acidocalcisome fraction have 50% more PPase activity than the yolk fraction (Figure 5B). To further investigate the PPi hydrolysis activity in the acidocalcisome-like organelles, membrane preparations of acidocalcisome and yolk fractions were obtained and tested in the presence of known inhibitors and co-factors of the V-H^+^-PPase. Results showed that AMDP (a non-hydrolyzable PPi analog which is considered as a specific inhibitor of V-H^+^-PPase) [44] was able to inhibit approximately 35%. of the enzyme activity in acidocalcisomes (Figure S1). In addition, as expected, no activity was detected in the absence of Mg^2+^ (co-factor of the enzyme) or PPi (substrate) (Figure S1). Poly P quantification showed higher amounts of short- and long-chain poly P in the acidocalcisomes when compared to the yolk fraction (Figure 5C). To investigate the localization of poly P and the V-H^+^-PPase in the acidocalcisome-like organelles, antibodies against the poly P binding domain (PPBD) of *S. cerevisiae* exopolyphosphatase (rScPPX1) with an *Xpress* epitope tag were used together with antibodies against the V-H^+^-PPase in immunofluorescence. Results showed that both poly P and the V-H^+^-PPase are found in the same vesicles present in the acidocalcisome enriched fraction (Figures 5D–G).

### Quantification of inorganic elements in the eggs and acidocalcisome fractions

Total egg homogenate (THE), yolk and acidocalcisome fractions were obtained, dried on speed-vac and the contents of phosphorus, calcium, magnesium and sodium were quantified by inductively coupled plasma optical emission spectroscopy. Results showed that the egg contains approximately seven times more phosphorus than calcium and magnesium, and large amounts of sodium (Table 1). Additionally, of the total amount of calcium in the eggs (18±0.6 µg), at least 24±0.9% (4.2±0.6) is present in the acidocalcisomes, suggesting that this is one of the main calcium storage compartments in the egg.

**Table 1:** Elemental quantification in the eggs using optical emission spectroscopy.

	Phosphorus	Calcium	Sodium	Magnesium
**Egg**	141±2.8	18±0.6	97±2.1	20±0.6
**Yolk**	138±4.6	13±0.6	95±5.2	18±0.6
**Accs**	3.8±1.0	4.2±0.9	0.11±0.01	0.31±0.1

Results are expressed in micrograms, as mean ± SD of three independent quantifications, from 165 mg of eggs (wet weight) which corresponds to 23±1.4 mg of dry weight or approximately 200 eggs.

### Acidification, poly P content and semi-quantitative X-ray elemental analysis of acidocalcisomes during early embryogenesis

**Figure 6:**
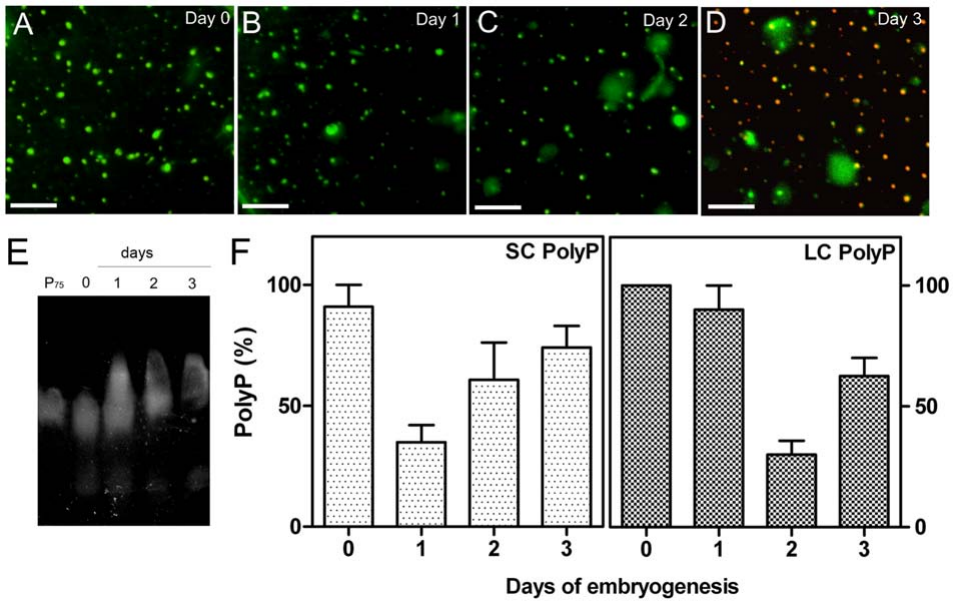
Acidocalcisomes during embryogenesis, acidification and Poly P quantification. Acidocalcisome fractions were obtained from different days of embryogenesis and incubated in the presence of 6 µM AO: **A,** day-0. **B,** day-1. **C,** day -2. **D,** day-3. Bars: 15 µm. **E,** agarose gel electrophoresis separation and poly P staining with DAPI. *Lane P75*: 5 µg of commercial Poly P_75+_; *Lanes 0, 1, 2* and *3* indicate days of development when poly P was extracted from acidocalcisomes and loaded into the gel. **F,** short- and long-chain poly P were quantified in the corresponding acidocalcisome fractions using the recombinant exopolyphosphatase of *S. cerevisiae*, as described under “Materials and Methods”. Error bars are mean ± S.E.M. (n = 3).

Observation of the acidocalcisome fractions obtained from eggs of different days of development in the presence of acridine orange (AO) allowed us to investigate the acidity of these organelles during early embryogenesis. This compound has been shown to be a reliable probe, both for spectrophotometric measurements and microscopic visualization of intracellular acidic compartments, including endosomes, lysosomes [44] and acidocalcisomes from different microorganisms [45] and insect eggs [6], [26]. Incubation of acidocalcisomes with AO showed that they appear as neutral organelles from day-0 to day-2 of embryogenesis, and that, at day-3, they become acidified (Figure 6, A–D), a period that coincides with the beginning of the mobilization of yolk proteins. The content of poly P in the acidocalcisome-like organelles during embryogenesis was detected by DAPI staining in agarose gels, evidencing the accumulation of this polymer with a shorter chain length smear in the lower part of the gel and a major spot with a slightly slower mobility than the poly P_75+_ standard. (Figure 6E). Additionally, quantification of the different chain-length poly P revealed that short-chain poly P levels decrease approximately 60% at day-1 and 30% and 15% at days 2 and 3, respectively, when compared to the levels of day-0 eggs (Figure 6F). Long-chain poly P levels remain unaltered at day-1, but decrease approximately 60% at day-2 and 30% at day-3, also when compared to day-0 eggs (Figure 6F). Semi-quantitative elemental analysis (Cliff-Lorimer ratios method) of the acidocalcisomal contents showed no relevant alterations in the relative levels of sodium, phosphorus, calcium, magnesium and potassium during the first 5 days of embryogenesis (tables S1 and S2).

## Discussion

In insects, as the embryogenesis occurs separately from the maternal body, the embryo development relies entirely on the yolk nutritional reserves. Mobilization of the yolk components to the embryo cells is, therefore, indispensable for egg viability, and investigations on the cellular events involved in yolk degradation may reveal potential targets for interference in vector's reproduction.

The yolk of meroblastic eggs is a highly regulated and compartmentalized cellular domain, specialized in the storage of reserve components for embryo development. It enables the yolk proteins, which are compartmentalized within individual and varied stores, to be hydrolyzed in a programmed time frame, and nourishes the embryo with fundamental molecules for cellular metabolism and growth. However, regardless of its importance for embryo development, the functional roles of the different yolk organelles and their interaction with the embryo cells during embryogenesis are still to be elucidated. In this context, we report here the characterization of acidocalcisome-like organelles in the yolk of the insect *R. prolixus*. The existence of these organelles in the eggs was confirmed by observations that some small vesicles in the yolk share many characteristics with acidocalcisomes, such as: 1) high electron density when observed by transmission electron microscopy as a whole mount; 2) large amounts of calcium and other cations, such as magnesium, sodium and potassium 3) large amounts of phosphorus in the form of poly P; 4) an empty vacuolar appearance in thin sections examined by standard TEM; 5) the presence of a V-H^+^-PPase in their enclosing membrane; and 6) the ability to undergo acidification, being able to accumulate acidophilic dyes such as AO.

The presence of a V-H^+^-PPase in acidocalcisomes has been extensively reported [6], and detection of a membrane bound PPase activity in yolk vesicles has been already described by our group in the insects *R. prolixus* [6] and *Periplaneta americana* [27]. In the latter, the enzyme was also localized to small vesicles similar to acidocalcisomes [27]. In this work, the PPase activity previously detected in *R. prolixus* eggs was shown to be associated to the acidocalcisomes, and showed similar characteristics to those previously described for the yolk vesicles [6], plants, bacteria and protists [46]. Additionally, the enzyme was shown to be associated with the acidocalcisome membrane and to be moderately inhibited by AMDP (an inhibitor of V-H^+^-PPases, [47]). The V-H^+^-PPase was proposed to be an alternative way of generating a proton electrochemical gradient across the membrane of organelles during germination in plants [46]. It could have a similar role during embryo development, which also involves massive synthesis and degradation of macromolecules necessary for rapid embryo growth [48]. Acidification of the egg compartments is indispensable for proper development and the existence of such an alternative proton pump seems a convenient attribute of the insect yolk.

The egg cortex is a large assemblage of the plasma membrane to which a characteristic set of cytoskeletal elements, organelles and macromolecules adhere. It is possible to isolate the egg cortex of echinoderms, molluscs, mouse and *Xenopus laevis*, but the present knowledge of cortex ultrastructure and function is relatively limited, compared to the extensive data available for plasma membrane permeability and calcium release [49], [50]. In meroblastic eggs, found in most insects, the egg cortex is the region where the cleavage nuclei migrate and the first cellularization of the embryo is achieved during early embryogenesis. Thus, localization of the acidocalcisomes in the egg cortex suggests that the egg periphery is also a region of the cell of particular importance in insect eggs, and might contain important organelles and macromolecules necessary for patterning formation and/or regulation of yolk mobilization to the newly formed embryo cells. In fact, it has been shown that the endoplasmic reticulum of *R. prolixus* is also found in the egg periphery during early embryogenesis and that the peripheral ooplasm also contains a varied set of vesicles and mitochondria (differently from the bulk cytoplasm which is mostly filled with large yolk granules) [51].

Inorganic elements are fundamental for eukaryotic cell metabolism and comprise bulk biological elements (present in large amounts in the cell, e.g., calcium and phosphorus) and trace elements (e.g., magnesium, zinc and manganese). They are known to be essential as co-factors for many enzymes that play important roles in tissue growth, electron-transfer processes and protein synthesis [52], [53]. In this regard, these components must be taken into account in the yolk storage reserve, especially in a system of intense biosynthetic reactions as a growing embryo. Most of the efforts in studies about the yolk components were directed to unveiling the role and isolation of specific macromolecules, mainly the yolk proteins [54]–[56]. It is widely reported in the literature that vitellins from insects are phosphorylated, and can bind cations such as Ca^2+^ and Mg^2+^ [57], [58]. However, it is still uncertain whether or not the products of vitellins hydrolysis would provide sufficient amounts of the inorganic elements required for the growing embryo, mostly for bulk biological elements that are needed in greater amounts by the cells. In this context, the acidocalcisomes might be the compartment in the yolk that provides inorganic elements for the embryo.

Regarding phosphorus storage, poly P is known to be an anionic polymer that can bind to several cations and a Pi store in different organisms [58]–[60]. The presence of poly P stored in acidocalcisomes has been extensively reported in different models [15], [23], and changes in its levels according to varied extracellular stimuli were observed [35]. Interestingly, the levels of poly P in *Rhodnius* acidocalcisomes tend to decrease slightly before the beginning of yolk mobilization, and a decrease in the levels of this polymer was already observed in eggs of the cattle tick *Boophilus microplus* [61] and the cockroach *P. americana* [37]. These data suggest that poly P might be widely used as Pi source for growing embryos, mainly in invertebrates, that do not store massively phosphorylated yolk proteins as phosvitins, known in vertebrate models [62]. In addition to its possible role in the storage of Pi, poly P polymers in the eggs of *R. prolixus* were already characterized and shown to inhibit the activity of an aspartic protease (known to target the yolk proteins in this insect) in a dose dependent manner [63]. Thus, poly P degradation in the acidocalcisomes during embryogenesis could also work as an additional step in regulation of the degradation of yolk proteins, concurrently with the acidification of the yolk granules via proton pumps, which results in activation of the acid hydrolases.

The fact that the acidocalcisomes store at least 24% of the total calcium detected in *R. prolixus* eggs indicates that this organelle is an important calcium storage compartment. It is known that most of the calcium present in acidic stores is probably bound to polyanionic matrixes [64]. Thus, stored poly P might represent the major Ca^2+^ buffer present in the acidocalcsiomes of *R. prolixus*, as it happens in acidocalcisomes from protozoan [15] and other eggs [26]–[28]. Calcium plays essential roles during embryonic development as second messenger for several signaling pathways [65], and global calcium release events are extensively described at fertilization and early steps of development [66]. In insects, events of calcium-modulated fusion of yolk organelles were described by our group, and shown to be important for yolk mobilization [67], [68]. Thus, we cannot rule out the possibility that acidocalcisomes can participate in such events of calcium release and re-uptake, perhaps combined with the peripheral endoplasmic reticulum via calcium induced calcium release (CICR). Another possibility is that most of the calcium present in the acidocalcisomes is not available for signaling, being bound to poly P or complexed with phosphorus in inorganic minerals as calcium phosphates. In this case, calcium from the acidocalcisomes could be used as an inorganic element supply for the embryo cells in later steps of embryogenesis, when the cell mass of the embryo is increased concomitantly with the consumption of fundamental molecules. It is still uncertain if the calcium stored in the acidocalcisomes could be used as a supply of inorganic elements for the embryo, as second messenger available for calcium signaling, or as both. Nevertheless, the fact that this element is stored in such large amounts in those organelles surely suggests an important function of the acidocalcisomes in the homeostasis of this ion during embryogenesis.

Taken together, results show that acidocalcisomes in the eggs may play multiple roles during embryogenesis, and can be seen as essential components of the yolk in insect eggs. These organelles may function as storage compartments for inorganic elements to the embryo cells, and cooperate in different aspects of the regulation of the yolk degradation. Acidocalcisomes may participate in the regulation of yolk proteolysis either as a Ca^2+^ storage compartment for intracellular signaling, which is possibly important for the fusion events between the yolk organelles [67], [68], or as poly P storage, which might work as an additional modulator of proteolytic activity [63] (Figure S2). Evidences are, therefore, that regulation of yolk degradation in insects is coordinated in several steps, and that acidocalcisomes are likely to be player organelles in this process.

## Supporting Information

Figure S1 **PPi hydrolysis activity in acidocalcisomes is sensitive to specific inhibitors.** PPi hydrolysis (PPase) activity was measured in membranes of the yolk and acidocalcisome fractions (accs). AMDP (40 µM) was added where indicated. Data are from 4 experiments, and show means ± S.E.M. (*) indicates significant differences (one way ANOVA, p<0.05).
(TIF)

Figure S2 **Schematic representation of the potential functional roles of acidocalcisomes during insect embryogenesis.** Acidocalcisomes are likely to work as storage compartments of inorganic elements to the embryo cells, cooperating in other aspects related to regulation of the yolk degradation. Ca^2+^ in the acidocalcisomes may be used as second messenger for intracellular signaling during early embryogenesis, and poly P polymers can be used as Pi source for the embryo cells and regulators of the aspartic protease activity.
(TIF)

Table S1 **Relative elemental quantification in the acidocalcisomes during early embryogenesis (Cliff-Lorimer method).** Semi quantitative X-ray microanalyses of the acidocalcisomes at different days of embryogenesis in eggs of *R. prolixus*. Numbers are expressed as the atomic % of each element (mean ± SEM, n_accs_ = 7).
(DOC)

Table S2 **Relative elemental quantification in the acidocalcisomes during early embryogenesis relative to phosphorus signal.** Semi quantitative X-ray microanalyses of the acidocalcisomes in different days of embryogenesis in eggs of *R. prolixus*. Numbers indicate the % of the ions signal relative to the phosphorus signal (mean ± SEM, n_accs_ = 7).
(DOC)

Movie S1 Confocal laser scanning microscopy reconstruction of a thick longitudinal section of the egg showing the strong autofluorescence in the chorion in the control experiment for V-H^+^-PPase localization.
(AVI)

Movie S2 Confocal laser scanning microscopy reconstruction of a thick longitudinal section of the egg showing the strong autofluorescence in the chorion and the V-H^+^-PPase localization in the egg periphery.
(AVI)
