# Supplementary figures and images for: Acidocalcisomes as Calcium- and Polyphosphate-Storage Compartments during Embryogenesis of the Insect Rhodnius prolixus Stahl
Source: PLoS One. 2011 Nov 11;6(11):e27276. doi: 10.1371/journal.pone.0027276 (PMC3214050; doi:10.1371/journal.pone.0027276)

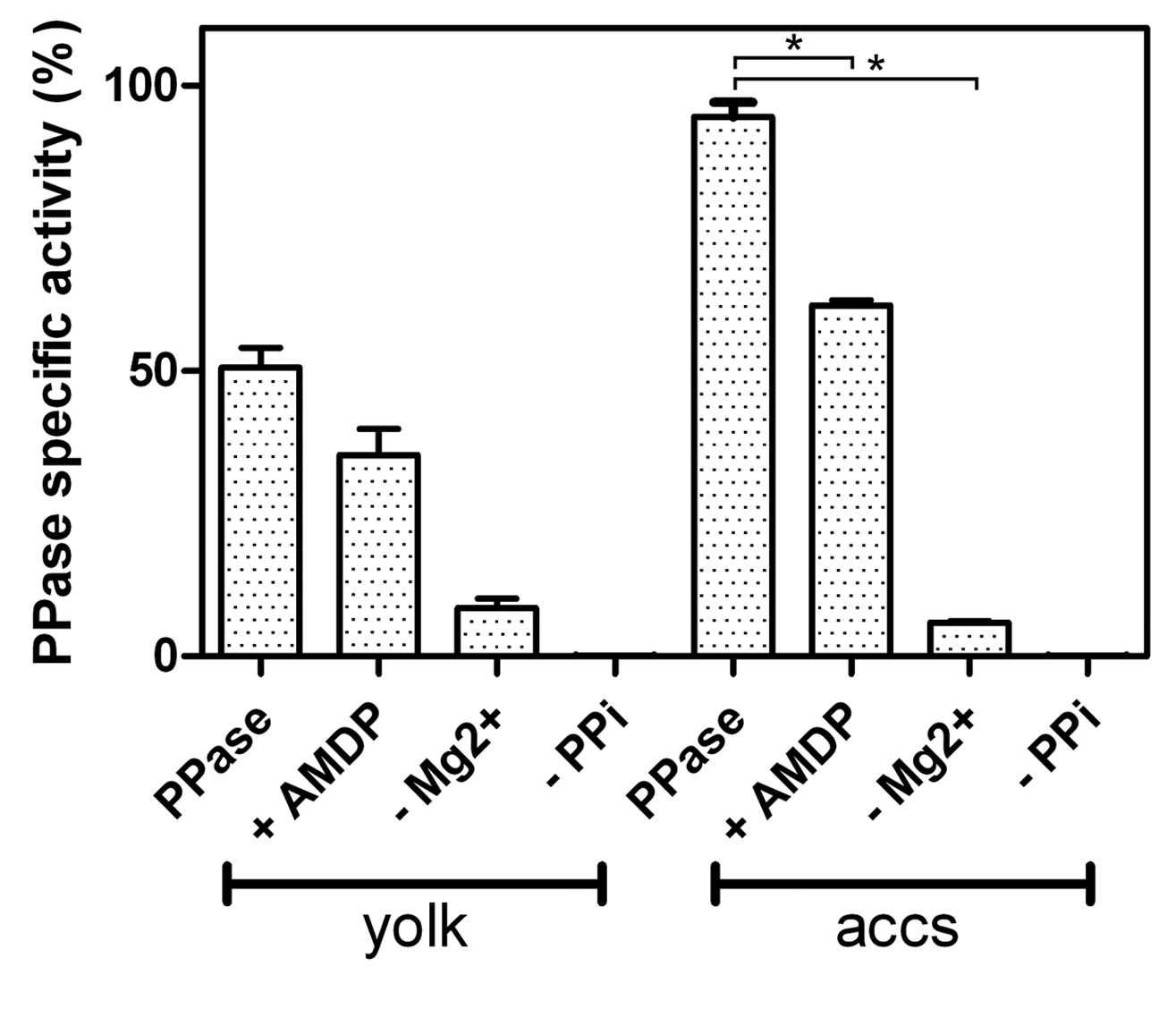

Supplement: Figure S1 — PPi hydrolysis activity in acidocalcisomes is sensitive to specific inhibitors. PPi hydrolysis (PPase) activity was measured in membranes of the yolk and acidocalcisome fractions (accs). AMDP (40 µM) was added where indicated. Data are from 4 experiments, and show means ± S.E.M. (*) indicates significant differences (one way ANOVA, p<0.05). (TIF) [file pone.0027276.s001.tif]

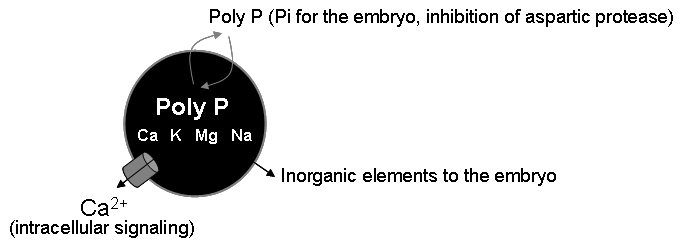

Supplement: Figure S2 — Schematic representation of the potential functional roles of acidocalcisomes during insect embryogenesis. Acidocalcisomes are likely to work as storage compartments of inorganic elements to the embryo cells, cooperating in other aspects related to regulation of the yolk degradation. Ca2+ in the acidocalcisomes may be used as second messenger for intracellular signaling during early embryogenesis, and poly P polymers can be used as Pi source for the embryo cells and regulators of the aspartic protease activity. (TIF) [file pone.0027276.s002.tif]
