# Supplementary material for: Acidocalcisomes as Calcium- and Polyphosphate-Storage Compartments during Embryogenesis of the Insect Rhodnius prolixus Stahl
Source: PLoS One. 2011 Nov 11;6(11):e27276. doi: 10.1371/journal.pone.0027276 (PMC3214050; doi:10.1371/journal.pone.0027276)
Supplement: Table S1 — Relative elemental quantification in the acidocalcisomes during early embryogenesis (Cliff-Lorimer method). Semi quantitative X-ray microanalyses of the acidocalcisomes at different days of embryogenesis in eggs of R. prolixus. Numbers are expressed as the atomic % of each element (mean ± SEM, naccs = 7). (DOC) [file pone.0027276.s003.doc]

Relative elemental quantification in the acidocalcisomes during early embryogenesis (Cliff-Lorimer method)

| **Days of development** | **Magnesium** | **Phosphorus** | **Chloride** | **Potassium** | **Calcium** |
| --- | --- | --- | --- | --- | --- |
| **0** | 3.4 ± 0.5 | 44.3 ± 2.4 | 2.4 ± 1.2 | 14.7 ± 0.8 | 35.1 ± 0.7 |
| **1** | 4.4 ± 0.3 | 46.6 ± 2.2 | 1.3 ± 0.8 | 12.9 ± 2.2 | 34.7 ± 2.6 |
| **2** | 5.6 ± 1.2 | 45.6 ± 1.2 | 1.7 ± 1.1 | 14.9 ± 2.0 | 32.1 ± 3.3 |
| **3** | 4.9 ± 0.6 | 46.2 ± 1.3 | 1.4 ± 0.6 | 6.3 ± 1.1 | 41.1 ± 1.1 |
| **4** | 4.7 ± 0.6 | 45.8 ± 1.6 | 0.9 ± 0.5 | 6.2 ± 2.7 | 42.3 ± 2.0 |
| **5** | 4.4 ± 0.9 | 42.4 ± 1.2 | 1.9 ± 0.5 | 14.2 ± 3.7 | 36.9 ± 3.6 |
| **Non fertilized** | 4.5 ± 0.7 | 46.81 ± 1.6 | 1.2 ± 0.4 | 9.1 ± 2.4 | 44.3 ± 3.2 |
