# Supplementary material for: Acidocalcisomes as Calcium- and Polyphosphate-Storage Compartments during Embryogenesis of the Insect Rhodnius prolixus Stahl
Source: PLoS One. 2011 Nov 11;6(11):e27276. doi: 10.1371/journal.pone.0027276 (PMC3214050; doi:10.1371/journal.pone.0027276)
Supplement: Table S2 — Relative elemental quantification in the acidocalcisomes during early embryogenesis relative to phosphorus signal. Semi quantitative X-ray microanalyses of the acidocalcisomes in different days of embryogenesis in eggs of R. prolixus. Numbers indicate the % of the ions signal relative to the phosphorus signal (mean ± SEM, naccs = 7). (DOC) [file pone.0027276.s004.doc]

Relative elemental quantification in the acidocalcisomes during early embryogenesis relative to the phosphorus signal (Cliff-Lorimer method)

| **Days of development** | **Magnesium** | **Chloride** | **Potassium** | **Calcium** |
| --- | --- | --- | --- | --- |
| **0** | 7.8 ± 1.3 | 5.6 ± 3.1 | 33.3 ± 3.6 | 79.3 ± 5.7 |
| **1** | 9.6 ± 1.1 | 2.9 ± 1.8 | 27.7 ± 5.1 | 74.5 ± 8.2 |
| **2** | 12.4 ± 2.8 | 3.8 ± 2.7 | 32.8 ± 5.3 | 70.3 ± 6.1 |
| **3** | 10.7 ± 1.3 | 2.9 ± 1.4 | 13.9 ± 2.7 | 89.2 ± 3.4 |
| **4** | 10.3 ± 1.0 | 1.9 ± 1.0 | 13.8 ± 6.4 | 92.4 ± 5.3 |
| **5** | 10.4 ± 2.3 | 4.5 ± 1.1 | 23.6 ± 9.1 | 87.0 ± 9.4 |
| **Non fertilized** | 9.9 ± 1.2 | 2.4 ± 1.0 | 11.9 ± 5.4 | 92.6 ± 9.0 |
